# Biochemical pathways supporting beta-lactam biosynthesis in the springtail *Folsomia candida*

**DOI:** 10.1242/bio.019620

**Published:** 2016-10-28

**Authors:** Wouter Suring, Janine Mariën, Rhody Broekman, Nico M. van Straalen, Dick Roelofs

**Affiliations:** Department of Ecological Science, Vrije Universiteit Amsterdam, De Boelelaan 1085-1087, Amsterdam 1081 HV, The Netherlands

**Keywords:** Beta-lactam, L-α-aminoadipate, Heat shock, Collembola, Gene expression

## Abstract

Recently, an active set of beta-lactam biosynthesis genes was reported in the genome of the arthropod springtail *Folsomia candida* (Collembola). Evidence was provided that these genes were acquired through horizontal gene transfer. However, successful integration of fungal- or bacterial-derived beta-lactam biosynthesis into the metabolism of an animal requires the beta-lactam precursor L-α-aminoadipic acid and a phosphopantetheinyl transferase for activation of the first enzyme of the pathway, δ-(L-α-aminoadipoyl)-L-cysteinyl-D-valine synthetase (ACVS). In this study, we characterized these supporting pathways and their transcriptional regulation in *F. candida*. We identified one phosphopantetheinyl transferase and three pathways for L-α-aminoadipic acid production, distinct from the pathways utilized by microorganisms. We found that after heat shock, the phosphopantetheinyl transferase was co-regulated with ACVS, confirming its role in activating ACVS. Two of the three L-α-aminoadipic acid production pathways were downregulated, while PIPOX, an enzyme participating in the pipecolate pathway, was slightly co-regulated with ACVS. This indicates that L-α-aminoadipic acid may not be a limiting factor in beta-lactam biosynthesis in *F. candida*, in contrast to microorganisms. In conclusion, we show that all components for L-α-aminoadipic acid synthesis are present and transcriptionally active in *F. candida*. This demonstrates how springtails could have recruited native enzymes to integrate a beta-lactam biosynthesis pathway into their metabolism after horizontal gene transfer.

## Abstract (precis)

**Summary:** Analysis of phosphopantetheinyl transferases and metabolic pathways for L-α-aminoadipic acid that are required for beta-lactam biosynthesis in the arthropod *Folsomia candida*.

## INTRODUCTION

Beta-lactam antibiotics are currently the most widely used antimicrobial compounds. Biosynthesis of these compounds has been described in several species of Actinobacteria and Proteobacteria and in several fungi (Ozcengiz and Demain, 2013). It was not reported in animals until recently, when the first metazoan beta-lactam biosynthesis genes were characterized in the springtail *Folsomia candida* (Roelofs et al., 2013). *Folsomia* *candida* is a soil-dwelling basal hexapod, the closest relatives of the insects (Misof et al., 2014). This animal contains a gene encoding a δ-(L-α-aminoadipoyl)-L-cysteinyl-D-valine synthetase (ACVS), as well as a functional isopenicillin N synthase (IPNS) enzyme that catalyzes the formation of the beta-lactam ring structure from the product of ACVS activity. Beta-lactam biosynthesis genes are involved in the stress response of *F. candida* and are upregulated upon exposure to a variety of stressors (de Boer et al., 2015). Most probably, *F. candida* acquired its beta-lactam genes through horizontal gene transfer (Roelofs et al., 2013). However, in addition to the core biosynthesis pathway, other pathways are required for beta-lactam biosynthesis.

In the first step of penicillin and cephalosporin biosynthesis, the tripeptide ACV is synthesized from L-cysteine, L-valine, and L-α-aminoadipic acid by ACVS, an enzyme belonging to the class of nonribosomal peptide syntethases (NRPSs). Like other NRPSs, ACVS requires its peptide carrier protein domains to be phosphopantetheinylated by a 4′-phosphopantetheinyl transferase (PPTase) to convert them to their active configuration (Wu et al., 2012). Only in its active form can ACVS bind the amino acids required for ACV biosynthesis. PPTases also occur in organisms without nonribosomal peptide synthetases because they are essential for phosphopantetheinylation of acyl carrier proteins involved in fatty acid metabolism (Bhaumik et al., 2005). It is known that metazoan PPTases can have broad substrate specificity and can even activate prokaryotic nonribosomal peptide synthetases in some cases (Joshi et al., 2003).

One of the substrates of ACVS is the nonproteinogenic amino acid L-α-aminoadipic acid (L-AAA). It serves as a building block for nonribosomal peptides, and also functions as an intermediate in lysine metabolism. Both beta-lactam-producing bacteria and fungi utilize specialized pathways for the production of L-AAA. Although all fungi produce L-AAA as an intermediate in a fungal-specific lysine biosynthesis pathway (Xu et al., 2006), fungi that synthesize beta-lactams utilize a second pathway in which lysine is catabolized to L-AAA using a ω-aminotransferase (Naranjo et al., 2005). Similarly, while most bacteria cannot synthesize L-AAA (Neshich et al., 2013), beta-lactam-producing bacteria can break down lysine to produce L-AAA using the enzymes lysine-6-aminotransferase and piperideine-6-carboxylate dehydrogenase (de La Fuente et al., 1997; Madduri et al., 1989).

For its beta-lactam biosynthesis pathway to be functional, *F. candida* needs to synthesize L-AAA. Three pathways have been described in animals in which L-AAA is an intermediate. Humans express two lysine degradation pathways in which L-AAA is produced as an intermediate: the saccharopine pathway and the pipecolate pathway (Hallen et al., 2013). L-AAA also functions as an intermediate in a third degradation pathway starting from 5-hydroxy-L-lysine (Veiga-da-Cunha et al., 2012). In the case of arthropods, the saccharopine pathway is present in all model insect species, while the pipecolate pathway is present in multiple species of Ecdysozoa, but appears absent in insects.

While supporting pathways for beta-lactam biosynthesis have been well-studied in microorganisms, it remains unknown how these pathways are organized in the springtail *F. candida*. This study aims at unraveling genes involved in L-AAA metabolism and phosphopantetheinylation in *F. candida* and their transcriptional regulation. To that end, we analyzed the transcriptome of *F. candida* and reconstructed full-length cDNAs for L-AAA metabolic pathways and PPTases. We know from previous experiments that the beta-lactam biosynthesis pathway of *F. candida* is induced during stress events. Here we hypothesize that supporting pathways will be co-regulated with the expression of beta-lactam biosynthesis genes during stress.

## RESULTS

### L-α-aminoadipic acid metabolism in *F. candida*

**Fig. 1.**
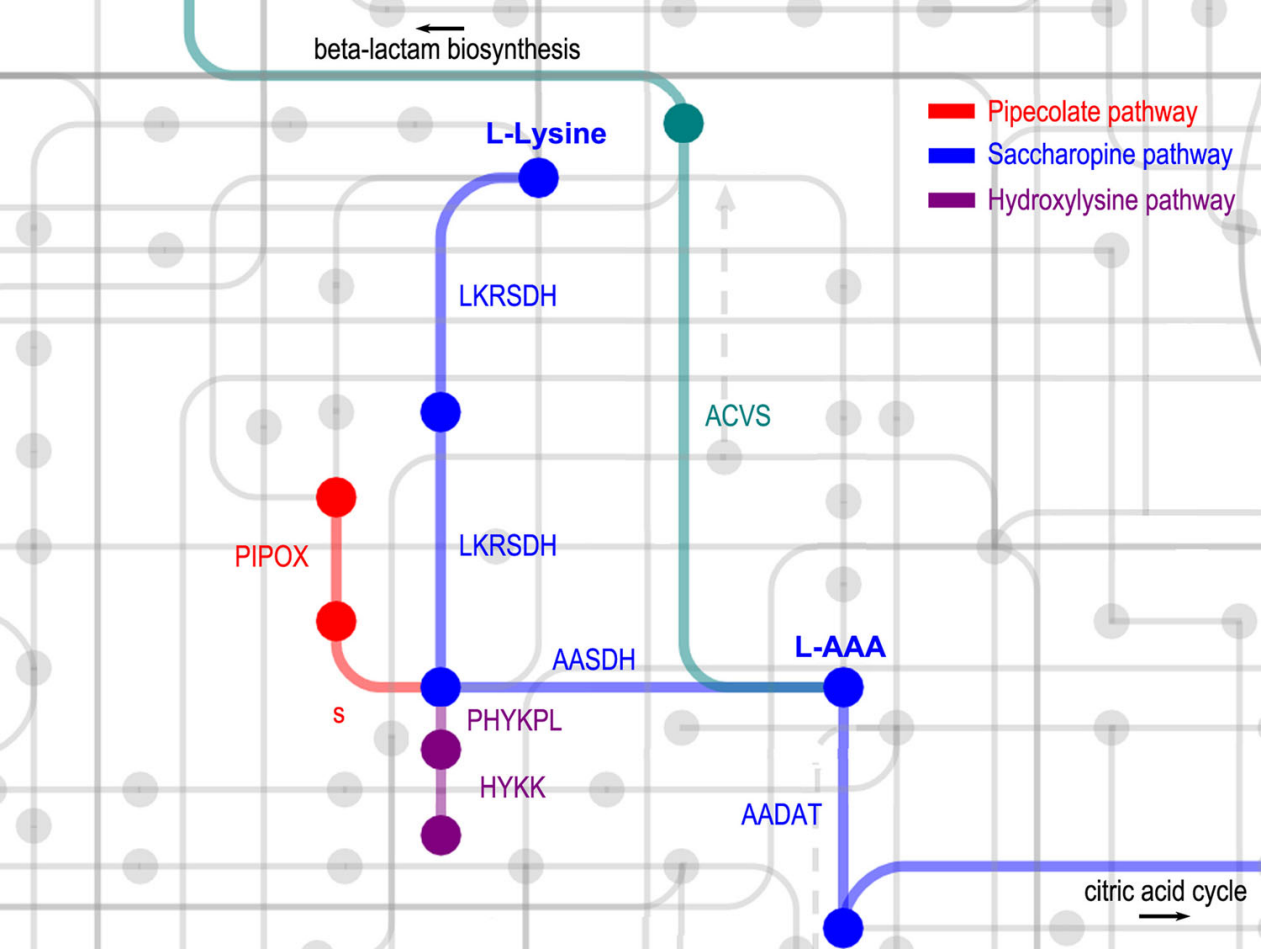
**L-α-aminoadipic acid metabolism in *F. candida*.** The saccharopine pathway (blue) consists of a bifunctional lysine-ketoglutarate reductase/saccharopine dehydrogenase (LKRSDH), L-aminoadipate-semialdehyde dehydrogenase (AASDH), and kynurenine/2-aminoadipate aminotransferase (AADAT). The pipecolate pathway (red) consists of pipecolic acid oxidase (PIPOX) before it joins the saccharopine pathway. The hydroxylysine pathway (magenta) consists of hydroxylysine kinase (HYKK) and 5-phosphonooxy-L-lysine phospho-lyase (PHYKPL). ‘s’ indicates a spontaneous (non-enzymatic) reaction. L-α-aminoadipic acid (L-AAA) is used in the first step of beta-lactam biosynthesis by δ-(L-α-aminoadipoyl)-L-cysteinyl-D-valine synthetase (ACVS). Figure produced using KEGG PATHWAY Database (http://www.genome.jp/kegg/pathway.html; Kanehisa et al., 2015).

Blast searches in combination with conserved domain searches using Pfam and KEGG (see Materials and Methods section) identified three metabolic pathways in *F. candida* in which L-AAA is an intermediate (Table 1). From the transcriptome data, we constructed the metazoan saccharopine and pipecolate pathways. In addition, the 5-hydroxy-L-lysine degradation pathway was identified. All three pathways converge at 2-aminoadipic semialdehyde which is converted to L-AAA by L-aminoadipate-semialdehyde dehydrogenase (Fig. 1). L-AAA is then available as a substrate for ACVS and thus supports beta-lactam biosynthesis. L-AAA can also be catabolized by kynurenine/2-aminoadipate aminotransferase and further converted to acetyl-CoA at which point it enters the citric acid cycle. The bacteria-specific L-AAA biosynthesis pathway consisting of lysine-6-aminotransferase (encoded by *lat*) and piperideine-6-carboxylate dehydrogenase (encoded by *pcd*), and the fungi-specific ω-aminotransferase encoded by *oat1* are not present in *F. candida*. Furthermore, we identified a single PPTase in *F. candida*, L-aminoadipate-semialdehyde dehydrogenase-phosphopantetheinyl transferase.

**Table 1.**
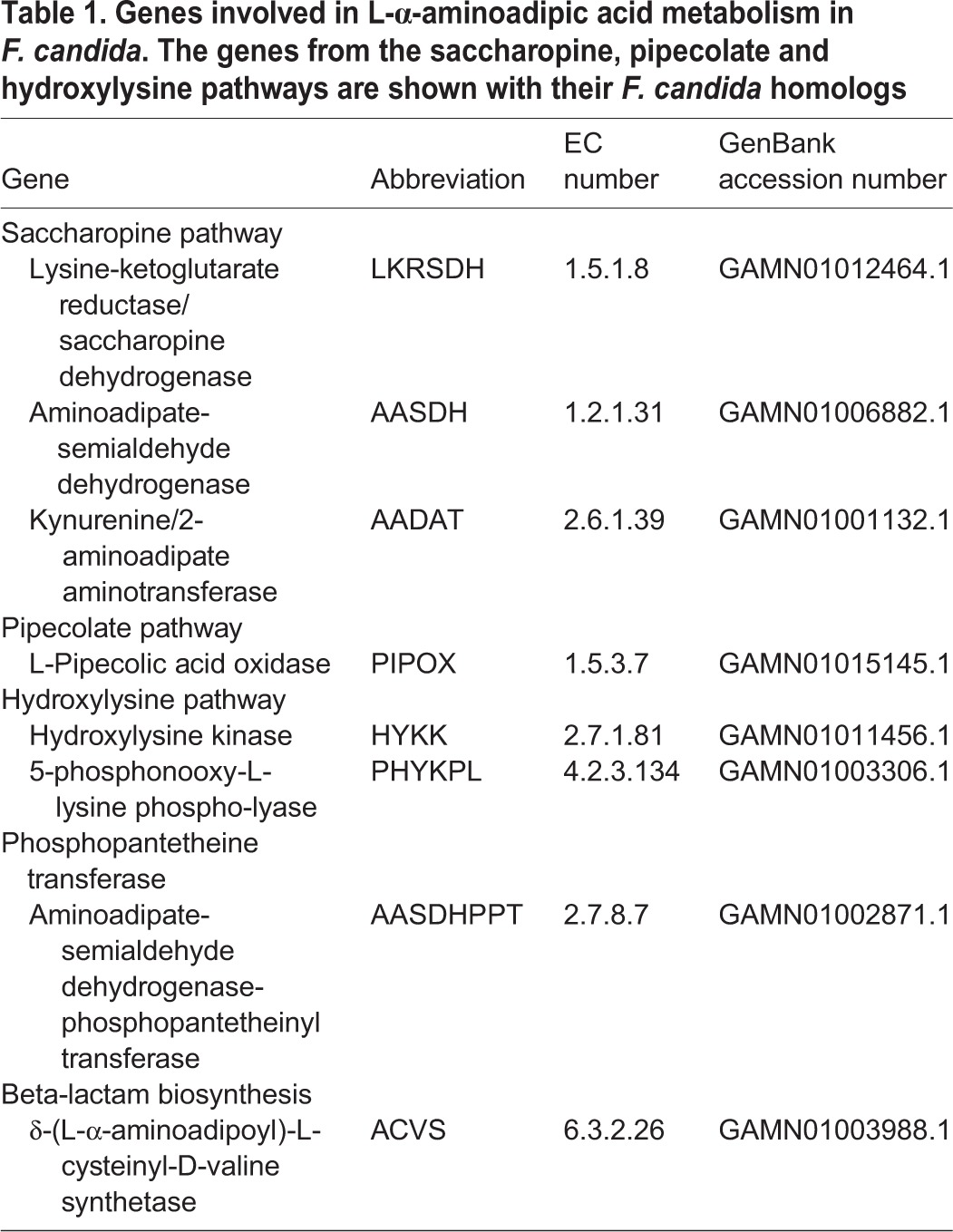
**Genes involved in L-α-aminoadipic acid metabolism in *F. candida*. The genes from the saccharopine, pipecolate and hydroxylysine pathways are shown with their *F. candida* homologs**

### Expression of ACVS and AASDHPPT

**Fig. 2.**
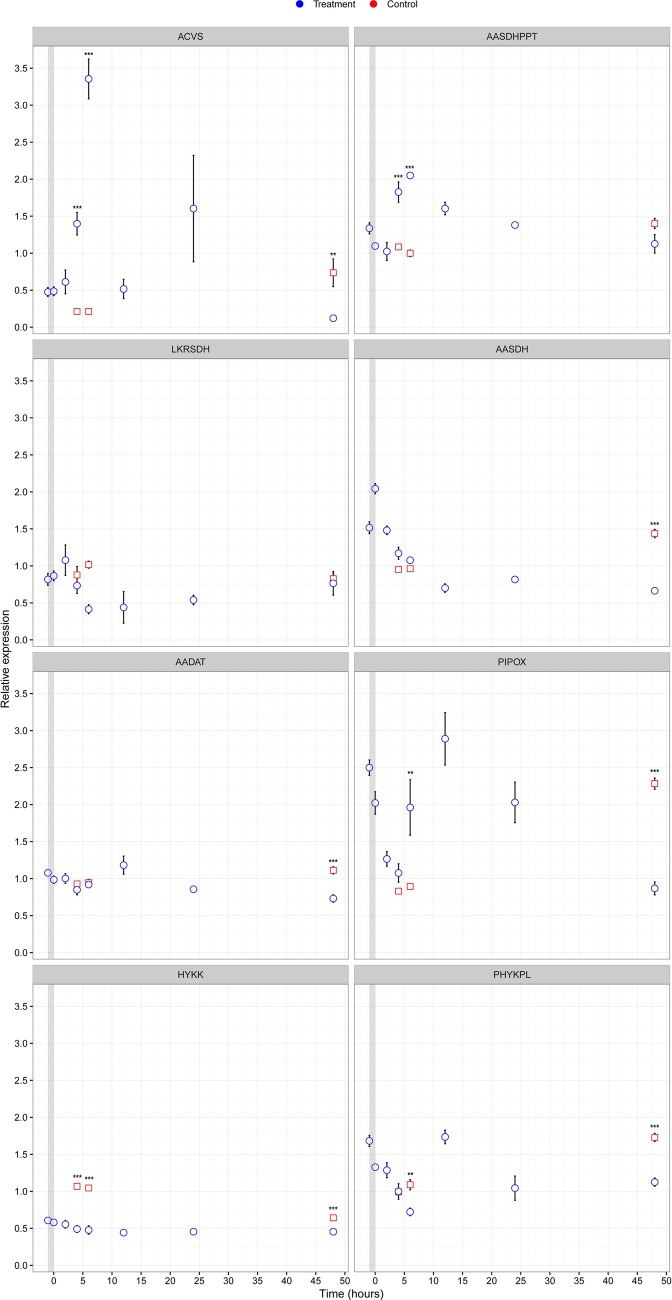
**Relative expression of supporting pathways for beta-lactam biosynthesis in *F. candida* after heat shock.** Gene expressions were determined from qPCR-derived cycle threshold values for cDNA amplification relative to two reference genes. Points depict observed means for each sampling time (*n*=4) and error bars depict the standard error of the means as a function of time. Blue bullets, samples that received heat shock; red squares, controls. The grey column represents the time of the heat shock. Asterisks depict significance level, (*P<0.05; **P<0.01; ***P<0.001) from the two-way ANOVA analysis in R 3.2.2.

Gene expression analysis using qPCR assays show that ACVS was significantly upregulated in response to heat shock (F test, *F*_2,23_=19.39, *P*<0.001) (Fig. 2). The expression reached its maximum at six hours after heat shock (mean fold-regulation, *M*=3.36, standard deviation, s.d.=0.54) and was over 15 times higher compared to controls (*M*=0.21, s.d.=0.051). At 48 h post heat shock, ACVS expression had returned to a low level (*M*=0.12, s.d.=0.07). The PPTase AASDHPPT showed a similar expression profile compared to ACVS reaching a maximum at the same time point (*F*_2,23_=54.05, *P*<0.001). Expression of the PPTase went up ∼twofold at 6 h after heat shock (*M*=2.1, s.d.=0.036) compared to controls (*M*=1.0, s.d.=0.089). In conclusion, both genes are significantly upregulated upon heat shock, with ACVS being the most inducible gene.

### Expression of L-α-aminoadipic acid metabolic genes

We obtained expression profiles of the three L-AAA metabolic pathways in *F. candida* that are described in Fig. 1. Expression of LKRSDH, the first gene of L-lysine degradation through the saccharopine pathway, was not significantly up- or downregulated after heat shock (*F*_2,23_=2.33, *P*=0.14). The second gene of the saccharopine pathway encodes AASDH, which is the enzyme that catalyzes the formation of L-AAA. This enzyme showed significant regulation in the time interval after heat shock (*F*_2,23_=144.4, *P*<0.001). More specifically, expression of AASDH at 48 h after heat shock (*M*=0.66, s.d.=0.07) was significantly lower compared to controls (*M*=1.44, s.d.=0.11). Expression of the gene encoding AADAT, the enzyme that catalyzes the degradation of L-AAA, was significantly downregulated after heat shock (*F*_2,23_=18.18, *P*<0.001) comparable to AASDH. Likewise, the expression of AADAT showed significantly lower transcript levels when compared to controls (*M*=1,1, s.d.=0.097) at 48 h after heat shock (*M*=0.73, s.d.=0.10).

A significant effect on expression of both HYKK (*F*_2,23_=44.49, *P*<0.001) and PHYKPL (*F*_2,23_=10.27, *P*<0.01) was observed in the 5-hydroxy-L-lysine pathway. We observed significant down regulation of PHYKPL at six hours after heat shock (*M*=0.72, s.d.=0.10) when compared to controls (*M*=1.09, s.d.=0.14). In control samples, HYKK expression increased after 4 h resulting in a significantly higher expression of this gene in controls (*M*=1.1, s.d.=0.023) compared to treated samples (*M*=0.49, s.d.=0.093). Expression of PIPOX, an enzyme participating in the pipecolate pathway, showed significant differential expression at two time points after heat shock (*F*_2,23_=29.86, *P*<0.001). First, it was induced up to twofold at 6 h after heat shock. Subsequently, significant down regulation was observed at 48 h post heat shock (*M*=0.89, s.d.=0.073) compared to controls (*M*=2.3, s.d.=0.75). In conclusion, synthesis of L-α-aminoadipic acid does not seem to be limiting during ACV synthesis in *Folsomia*, although there may be a slight preference for the pipecolate pathway.

## DISCUSSION

This work provides the first study on supporting pathways for beta-lactam biosynthesis in an animal. We identified and sequenced complete genes involved in the metabolism of the beta-lactam precursor L-AAA in the springtail *Folsomia candida* and showed that all components for the production of L-AAA are present and transcriptionally active. We found that ACVS and the PPTase AASDHPPT are co-regulated during stress, confirming a role for AASDHPPT in activation of ACVS. Previously, we applied *in situ* hybridization to show that ACVS is localized and strongly induced in the gut epithelium of the animal (Nota et al., 2008). Furthermore, Roelofs et al. (2013) showed that the formation of a beta-lactam can be catalyzed by beta-lactam biosynthesis genes belonging to the genome of *Folsomia*. Very recently, we detected and quantified beta-lactam compounds in *Folsomia* using an ELISA assay *in vivo*, and preliminary data show that this metabolite increases in abundance after heat shock treatment (W.S., unpublished data). As such, the current data provide important information on how beta-lactam biosynthesis is organized in *F. candida* and how native enzymes may have enabled *F. candida* to successfully integrate beta-lactam biosynthesis into its metabolism following horizontal gene transfer.

A PPTase is required for activation of ACVS, the first enzyme in beta-lactam biosynthesis, through the attachment of phosphopantetheine groups to its peptide carrier protein domains. We identified a single PPTase in *F. candida*, similar to other animals (Praphanphoj et al., 2001). Both ACVS and the PPTase AASDHPPT, were co-induced in response to heat shock. However, AASDHPPT only increased twofold, while ACVS activation reached over 15 times higher expression when compared to controls. This may suggest that the amount of PPTase is rate limiting in metabolite synthesis. A recent study measuring Michaelis–Menten kinetics (K_m_) of the NRPS bPCP domain from *Pseudomonas aeruginosa* and several PPTases from different unrelated prokaryotes indicate that the K_m_ of PPTases can be twice as high as K_m_s of NRPS domains (Owen et al., 2011). However, this would still not compensate for the discrepancy between the induction levels of ACVS and AASDHPPT. In bacteria, multiple PPTases are often identified which are utilized for activation of either nonribosomal peptide synthetases (NRPS) or fatty acid synthases (Lambalot et al., 1996; Mootz et al., 2001). Also, PPTases can exhibit broad substrate specificity. For example, *Homo sapiens* AASDHPPT can activate the prokaryotic NRPS tyrocidine synthetase of *Bacillus brevis* even though NRPSs are not present in humans (Joshi et al., 2003). Furthermore, the *Bacillus subtilis* PPTase Sfp exhibits broad substrate specificity and can phosphopantetheinylate ACVS of the fungus *Penicillium chrysogenum* (Gidijala et al., 2009). The co-regulation of AASDHPPT and ACVS during stress and the reported broad substrate specificity of PPTases support the notion that AASDHPPT activates ACVS in *F. candida*.

*F. candida* utilizes pathways for L-AAA production that are different from beta-lactam-producing microorganisms. The lysine degradation pathways of beta-lactam-producing fungi and bacteria are not present in *F. candida*. Instead, *F. candida* utilizes three metazoan degradation pathways in which L-AAA is an intermediate. The saccharopine pathway is present in many animals including humans and insects (Hallen et al., 2013; Wan et al., 2015), while the pipecolate pathway has been described in multiple metazoans including nematodes and chordates (Mihalik and Rhead, 1989; Umair et al., 2012). In addition, we found the recently characterized metazoan hydroxylysine degradation pathway (Veiga-da-Cunha et al., 2012) in *F. candida*. Beta-lactam biosynthesis gene clusters in bacteria include the *lat* gene for L-α-aminoadipic acid production (Coque et al., 1991; Madduri et al., 1991). The absence of this gene in *F. candida* indicates that it was either not part of the DNA that was horizontally transferred or subsequently degenerated after the horizontal gene transfer event.

While ACVS expression was low in *F. candida* under standard conditions, it was strongly upregulated under stress. The beta-lactam biosynthesis pathway is upregulated upon exposure to various stressors including cadmium (Nota et al., 2008), phenanthrene (Nota et al., 2009), and diclofenac (Chen et al., 2015) and seems to be part of the general stress response of *F. candida*. While ACVS was upregulated during stress, metabolism towards its substrate L-AAA was either slightly upregulated (PIPOX activation 6 h post heat shock) or downregulated in the case of the hydroxylysine pathway, indicating that L-AAA may not be a limiting factor in beta-lactam biosynthesis in *F. candida*. In *Streptomyces clavuligerus*, L-AAA is a limiting factor and overexpression of *lat* results in higher beta-lactam concentrations (Malmberg et al., 1993). However, the observed downregulation of lysine and hydroxylysine degradation in *F. candida* may also be more directly related to the effects of heat stress which could be more important for its survival than their role in beta-lactam biosynthesis.

As a result of the increased number of sequenced genomes, it has become clear that horizontal gene transfer has contributed to the biochemical diversification of many eukaryotic species (Boschetti et al., 2012; Crisp et al., 2015). Still, more barriers exist to the successful transfer of genes into the genomes of eukaryotes compared to transfer of genetic material between bacteria (Huang, 2013; Soucy et al., 2015). The transferred gene should end up in an expressed part of the genome, has to be incorporated behind a promoter, and should end up in the germ line and provide a fitness advantage to its new host. However, functional horizontal gene transfer can be further complicated by additional requirements for specific enzymes such as the need for helper enzymes and supporting biosynthetic pathways. Here we show that activating enzymes and supporting biosynthetic pathways necessary for beta-lactam production all exist in the springtail *F. candida*, providing a basis for further research into beta-lactam biosynthesis in this animal.

## MATERIALS AND METHODS

### Identification of PPTases and L-AAA metabolic pathways

We downloaded the *F. candida* transcriptome (PRJNA211850) from NCBI and used the Blast2GO suite version 3.1 (Gotz et al., 2008) for functional annotation. BlastX searches were performed against the NCBI non-redundant (nr) protein database (Pruitt et al., 2005) with an E-value threshold of 1e-05. Conserved domains were identified using InterProScan against the Pfam database (Finn et al., 2010) and Clusters of Orthologous Groups database at NCBI (Tatusov et al., 2001). Transcripts were annotated with enzyme commission (EC) numbers against the KEGG database (Kanehisa et al., 2004).

We compared the retrieved EC numbers and annotations to a selection of enzymes involved in the metabolism of L-AAA (Table S1). This selection included two L-lysine catabolic pathways, two L-lysine biosynthetic pathways, a 5-hydroxy-L-lysine catabolic pathway, and three genes found in beta-lactam-producing microorganisms: lysine-6-aminotransferase (*lat*), piperideine-6-carboxylate dehydrogenase (*pcd*), and ω-aminotransferase (*oat1*). Subsequently, all identified genes were validated by reciprocal blast searches with Swiss-Prot-curated L-AAA metabolism genes (Table S1) against the *F. candida* transcriptome with a threshold of 1e-05. Identification of PPTases was performed by searching for transcripts annotated with a 4′-PPTase domain (Pfam01648 or COG2091). Subsequently, all identified genes were used in qPCR analysis.

### Sequencing of genes involved in L-AAA metabolism

RNA was isolated from a stock culture of >28-day-old *F. candida* females (‘Berlin strain’; Vrije Universiteit Amsterdam) maintained as previously described (de Boer et al., 2009), using the SV RNA isolation kit (Promega). M-MLV reverse transcriptase (Promega) was used to produce cDNA according to the instructions of the manufacturer. In order to amplify the full-length open reading frames of genes involved in L-AAA metabolism, gene-specific primers were designed based on genomic data (Table S2). The amplified fragments were ligated into a pGEM-T vector (Promega) and transformed into *E. coli* DH5α according to the instructions of the manufacturer. The full-length ORFs were sequenced at Eurofins MWG Operon (Germany).

### Animal culture maintenance and heat shock

*Folsomia candida* were cultured in a 20°C climate room as previously described (de Boer et al., 2009). At the start of the experiment, adult *F. candida* (>28 days old) were divided over 60 plastic boxes (250 cm^3^) containing a layer of moist plaster of Paris mixed with charcoal. Next, animals were subjected to a heat shock in a 30.0±0.1°C water bath for 1 h before they were returned to the 20°C climate room. Control animals were treated the same way except that they were exposed to 20°C instead. Samples consisted of 75 pooled animals and were taken directly at heat shock and at 0, 2, 4, 6, 12, 24, and 48 h after heat shock. Four replicates were taken for each treatment and control groups at each time point. Animals were snap-frozen in liquid nitrogen and stored at −80°C until further processing.

### qPCR analysis

RNA was isolated using the SV RNA isolation kit (Promega) with an additional DNase treatment followed by phenol-chloroform purification and ethanol precipitation. DNA contamination was checked with a PCR, amplifying a fragment of IPNS. For reverse transcription, we used the M-MLV reverse transcription protocol of Promega. We used 5 µl of clean RNA in a 25 µl reaction volume. The cDNA was diluted five times before use in the qPCR assays. Primer sets were developed for the genes of interest (Table 2) according to de Boer et al. (2009). We normalized the input of RNA with two reference, succinate dehydrogenase complex subunit A (SDHA) and eukaryotic transcription initiation factor 1A (ETIF), that were previously shown to be the most stable during heat shock in *F. candida*, (de Boer et al., 2009).

**Table 2.**
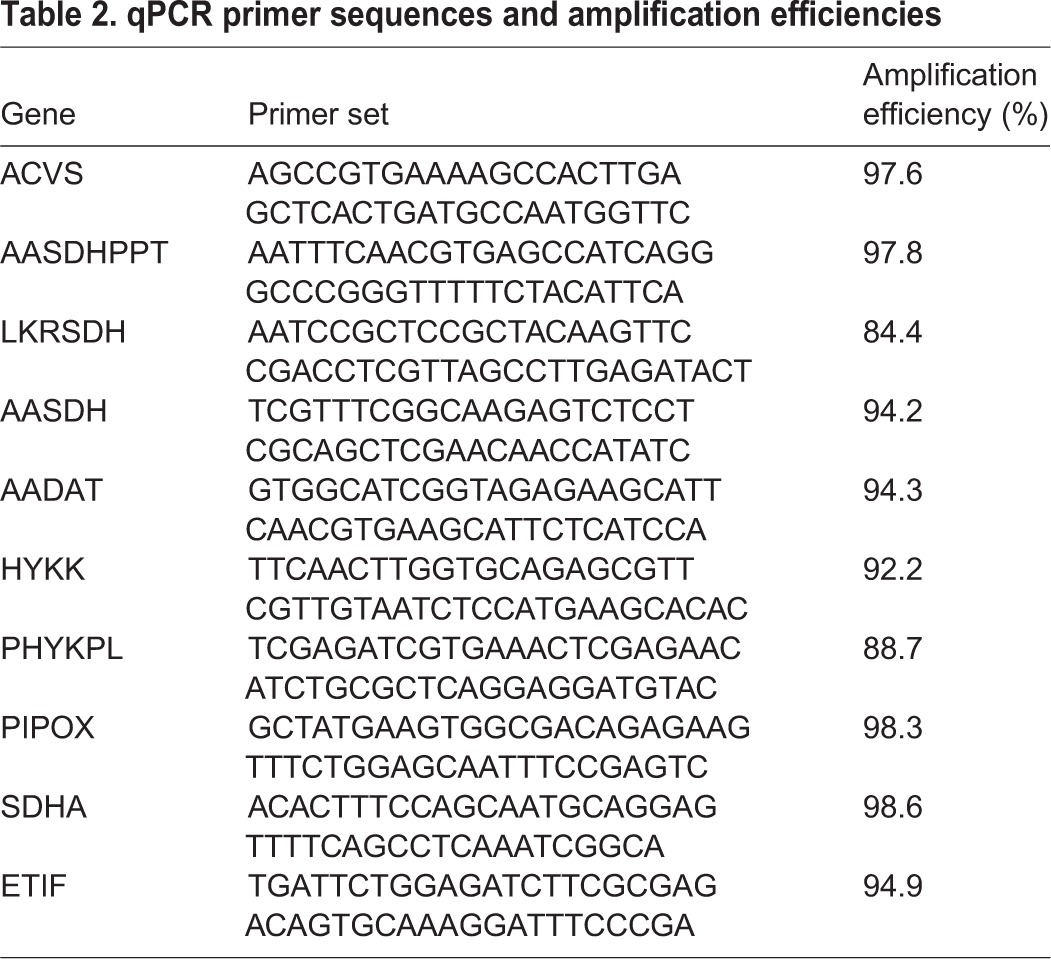
**qPCR primer sequences and amplification efficiencies**

For qPCR we used SensiMix SYBR No-ROX kit (BioLine), adding primers to a final concentration of 0.25 mM each. We used a two-step amplification protocol of 10 min. 95°C–40× (10 s. 95°C–30 s. 60°C) with a plate read after every cycle. After amplification, a melting curve was established (60°C–90°C, 0.5°C/read). qPCR runs were performed on a CFX Connect Real-Time PCR Detection System (Bio-Rad).

In order to determine PCR efficiencies, standard curves were obtained for the primer sets with six fourfold dilutions of a standard batch *F. candida* cDNA in duplicate (Pfaffl, 2001). Experimental cDNA samples were performed in duplicate for target genes and reference genes to yield cycle threshold values (C_t_). Plate results were collected in a single Gene Study data file and mean normalized expression values were calculated using the Bio-Rad CFX Manager software 3.1. Basic settings were manually adjusted, including primer efficiencies and baseline threshold, which was set to 1000 relative fluorescence units (RFU).

### Statistical analysis

Relative expression data were analyzed using a two-way ANOVA in R 3.2.2 (R Core Team, 2016) using time (three levels: 4, 6, and 48 h after heat shock) and treatments (two levels: control and heat-shocked) as factors. A Tukey HSD post hoc test was applied if the interaction between treatment and time was significant. Statistical differences were considered significant at *P*<0.05. QQ plots and box plots of the residuals were generated to check for normality and homogeneity of the data and a log transformation was applied when required.
